# Synergic Effect of N and Se Facilitates Photoelectric Performance in Co-Hyperdoped Silicon

**DOI:** 10.3390/nano14191591

**Published:** 2024-10-02

**Authors:** Haibin Sun, Xiaolong Liu, Caixia Xu, Long Xu, Yuwei Chen, Haima Yang, Xing Yang, Peng Rao, Shengli Sun, Li Zhao

**Affiliations:** 1 Key Laboratory of Intelligent Infrared Perception Chinese Academy Science (CAS), Shanghai Institute of Technical Physics, Chinese Academy Science (CAS), Shanghai 200043, China; sunhaibin007@gmail.com (H.S.);; 2 Department of Remote Sensing and Photogrammetry, Finnish Geospatial Research Institute, Vuorimiehentie 5, FI-02150 Espoo, Finland; 3 State Key Laboratory of Surface Physics, Department of Physics, Fudan University, Shanghai 200433, China; 4 Department of Electronics and Nanoengineering, Aalto University, Tietotie 3, FI-02150 Espoo, Finland; 5 School of Primary Education, Chongqing Normal University, Chongqing 400700, China; 6 Chongqing Key Laboratory of Micro&Nano Structure Optoelectronics, School of Physical Science and Technology, Southwest University, Chongqing 400715, China; 7 State Key Laboratory of Pulsed Power Laser Technology, Electronic Countermeasure Institute, National University of Defense Technology, Hefei 230037, China; 8 School of Optical-Electrical and Computer Engineering, University of Shanghai for Science and Technology, Shanghai 200093, China

**Keywords:** silicon, femtosecond (fs)-laser irradiation, N and Se hyperdoped silicon, photodetectors

## Abstract

Femtosecond-laser-fabricated black silicon has been widely used in the fields of solar cells, photodetectors, semiconductor devices, optical coatings, and quantum computing. However, the responsive spectral range limits its application in the near- to mid-infrared wavelengths. To further increase the optical responsivity in longer wavelengths, in this work, silicon (Si) was co-hyperdoped with nitrogen (N) and selenium (Se) through the deposition of Se films on Si followed by femtosecond (fs)-laser irradiation in an atmosphere of NF_3_. The optical and crystalline properties of the Si:N/Se were found to be influenced by the precursor Se film and laser fluence. The resulting photodetector, a product of this innovative approach, exhibited an impressive responsivity of 24.8 A/W at 840 nm and 19.8 A/W at 1060 nm, surpassing photodetectors made from Si:N, Si:S, and Si:S/Se (the latter two fabricated in SF6). These findings underscore the co-hyperdoping method’s potential in significantly improving optoelectronic device performance.

## 1. Introduction

Silicon-based photodetectors are widely used in various applications, including optical communication, imaging, sensing, and spectroscopy. Silicon-based photodetectors offer advantages such as high sensitivity, low noise, fast response times, and compatibility with existing silicon-based electronics technology, which have shown great compatibility with the current complementary metal–oxide–semiconductor (CMOS) circuits at a low cost [1,2,3,4,5]. However, the bandgap energy (1.12 eV) of silicon limits its application in photodetectors operating in longer infrared wavelengths >1.1 µm [6]. Hyperdoped silicon (hSi), synthesized by femtosecond (fs)-laser irradiation or ion implantation, exhibits strong broadband optical absorption, and a variety of photodetectors based on hSi operating at IR wavelengths have been fabricated [7,8,9]. Photodetectors based on sulfur-hSi (S-hSi) prepared in the atmosphere of SF6 exhibited amazing photoelectric responsivity [10,11]. Compared to the mainly used SF6 gas during fs-laser processing, the use of NF_3_ shows the following benefits: (1) NF_3_ is non-toxic and has a low environmental impact. (2) NF_3_ induced a much smoother spike surface, making it easier to deposit the electrode [12]. (3) N-hyperdoped Si (Si:N) fabricated from an fs-laser in NF_3_ has been proven to have better crystallinity with low structural defects [13,14,15]. However, the optical absorption, especially in the IR range, of Si:N fabricated with NF_3_ is not as high as Si:S with SF_6_ [16]. The latter also suffers from a drastic reduction in IR absorption after high-temperature rapid annealing treatments (RATs), which are typically used to repair laser-induced defects and to activate the dopants electrically [17,18]. Therefore, even combining both benefits from NF3 and SF_6_ by fabricating co-hyperdoped Si in a mixed gas atmosphere, the enhancement of photoelectric performance is still moderate [19]. Considering that selenium (Se), as a member of the same family of elements as S, shows higher optical absorption than S either with or without annealing [20,21,22,23], this motivates us to combine the high absorption from Se with better structural properties in an NF_3_ atmosphere.

In this article, we fabricate N- and Se-co-hyperdoped Si (Si:N/Se) by fs-laser irradiation of Si coated with the Se film precursor. We study the surface morphology, optical absorption, and Raman spectra resulting from the change in Se film thickness and laser fluence. We fabricate photodetectors based on the optimized Si:N/Se samples and characterize the photoelectric performance. To see the synergic effect of N and Se on the photoelectric performance, we also compare them with detectors fabricated from Si:N, Si:S, and Si:S/Se (S- and Se-co-hyperdoped Si), respectively.

## 2. Materials and Methods

In the fabrication process of co-hyperdoped silicon, a 250 µm n-type Czochralski (CZ) Si (111) wafer with a resistivity of 3–5 kΩ⸱cm was initially cut into pieces and cleaned using a standard RCA procedure. Subsequently, Se films of varying thicknesses (50, 70, and 100 nm) were thermally evaporated onto the Si surface to facilitate the doping process. A reference sample without the Se film was also prepared for comparison. The samples were then irradiated using a Yb:KGW fs-laser, which emitted a 1 kHz train of 190 fs laser pulses at a wavelength of 515 nm. This irradiation occurred in a 70 kPa atmosphere of NF_3_ or SF_6_ gases. The laser spot, with a diameter of 60 µm, was raster-scanned at a speed of 500 µm/s, treating an area of approximately 10 mm × 10 mm. Each spot on the wafer received an average of 350 laser shots. Additional information on the fs-laser doping procedure and experimental setup can be found in Ref. [8].

## 3. Results

**Figure 1:**
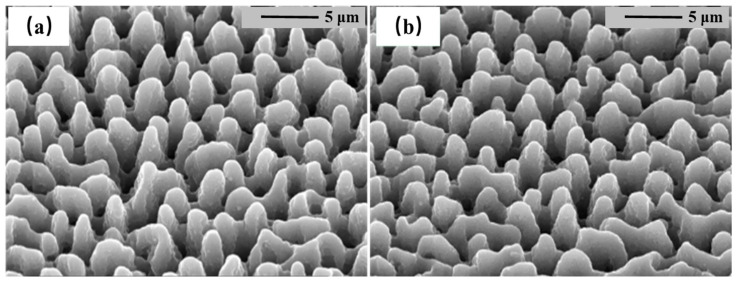
Scanning electron microscope (SEM) images of the (**a**) Si:N and (**b**) Si:N/Se at a laser fluence of 2.9 kJ/m2. The photos are viewed at 45° to the normal.

The surface morphology of the hSi samples shown in Figure 1 reveals spike-like microstructures present in both samples (Figure 1a,b) that exhibit consistent dimensions. These spike-like structures have a height ranging from 3 to 4 μm, a base width of 2 to 3 μm, and a spacing between structures measuring 2 to 3 μm. Despite introducing a 100 nm Se precursor in sample 1(b), there is no observable difference in the surface morphology compared to sample 1(a), which was fabricated without the Se precursor. This suggests that the presence of the Se film precursor does not alter the formation or characteristics of the spike-like microstructures on the hSi samples.

**Figure 2:**
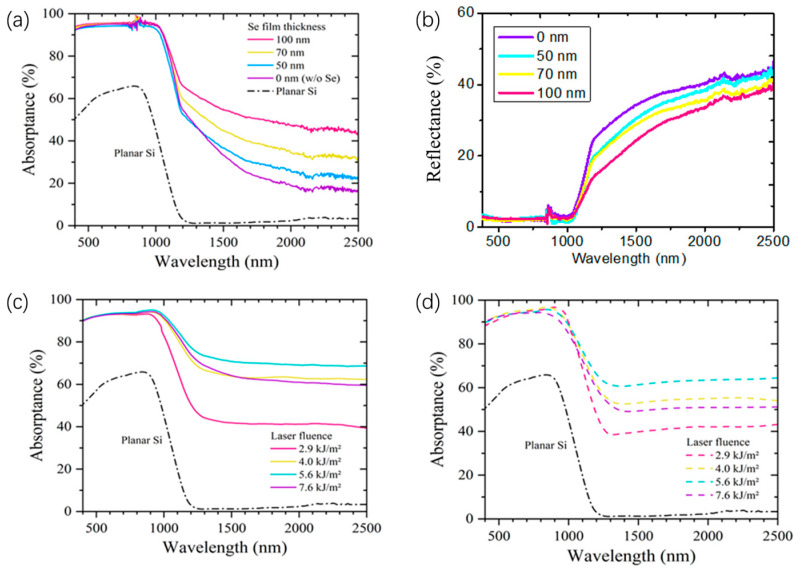
(**a**) Absorptance spectra of planar Si reference and Si:N/Se samples with different Se precursor thickness, with different laser fluence; (**b**) reflectance spectra of the Si:N/Se samples with different Se precursor thickness; absorptance spectra of planar Si reference and Si:N/Se samples with different Se precursor thickness (**c**) before and (**d**) after rapid thermal annealing at 800 K for 5 min.

The optical absorptance spectra, defined as A = 1 − R − T, were obtained by measuring the hemispherical reflectance (R) and transmittance (T) using a UV-vis-NIR spectrophotometer (Varian Cary 5 E) equipped with an integrating sphere. Figure 2a presents the absorptance of Si:N/Se samples fabricated with varying Se precursor film thicknesses, alongside Si:N and planar Si samples for comparative analysis. Notably, all hyperdoped samples exhibit absorptance exceeding 90% within the wavelength range of less than 1100 nm. To describe the effective absorption coefficient and thickness of the microstructure, we utilize the equation $\alpha^{\prime }=\frac{[1-R]\sum_{i}\langle l_{i}\rangle }{L}\alpha$, where $\sum_{i}\langle l_{i}\rangle$ is the effective thickness of the ablated microstructure, *α* is the absorption of light in the uniform Si plates, *L* is the thickness of the Si plates, *R* is the reflection of light, and *α****’*** is the effective absorption of light in the ablated microstructure. The multiple scattering of light within the structure can significantly enhance the microstructure’s effective thickness and absorption characteristics [24]. This phenomenon can potentially improve the photoresponse and absorption efficiency of the photodetectors utilized in this study. In the wavelength range of 1100–2500 nm, the Si:N sample exhibits an average absorptance of only 38%. However, this value increases for Si:N/Se samples as the Se precursor thickness is increased, indicating a higher Se doping concentration that contributes to enhanced sub-bandgap absorption. Figure 2b illustrates the reflectance spectra of the Si:N/Se samples as a function of Se precursor thickness. As the thickness of the Se precursor increases, the reflectance of the samples gradually decreases in the near- to mid-infrared region, correlating with the enhanced absorption observed in Figure 2a.

Figure 2c presents the absorptance of Si:N/Se samples with a 100 nm Se precursor after exposure to different laser fluences. The sub-bandgap absorptance increases with rising fluence, reaching an optimal value of 70% at approximately 5.6 kJ/m^2^, before subsequently decreasing. Generally, higher laser fluence enhances the doping concentration of impurity atoms, thereby increasing sub-bandgap absorptance. However, elevated laser fluence also leads to greater etching of surface materials, specifically the Se precursor in this case, which can reduce the doping concentration and, consequently, the sub-bandgap absorptance [25]. To activate the dopants following laser irradiation, the Si:N/Se samples underwent a rapid annealing treatment (RAT) in a nitrogen gas flow. Figure 2d displays the absorptance spectra of the samples corresponding to Figure 2c after annealing at 800 K for 5 min. The RAT process results in only a slight reduction in sub-bandgap absorptance, with the highest absorptance observed at approximately 65% at the optimal fluence of 5.6 kJ/m^2^ [26]. Notably, while a Si:S sample achieved over 90% sub-bandgap absorptance, this value drastically decreased to around 20% at the same annealing temperature in our previous work [12]. Furthermore, the Si:N/Se samples demonstrate more stable sub-bandgap absorptance compared to Si:S/Se samples subjected to similar annealing treatments [17]. The crystallinity of the Si:N/Se samples was characterized using a confocal Raman spectroscope excited by a 633 nm He-Ne laser. Previous studies have shown that fs-laser processing in NF_3_ results in significantly better crystallinity compared to samples fabricated in more commonly used atmospheres such as SF_6_ or N_2_, even without any subsequent annealing treatments [13,15].

**Figure 3:**
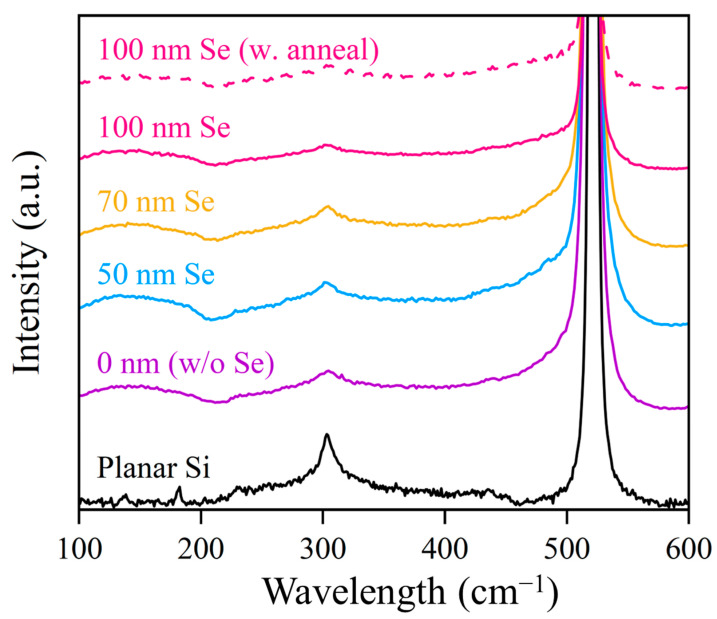
Raman spectra of unannealed planar Si reference and Si:N/Se samples with different Se precursor thicknesses. A Si:N/Se sample with a 100 nm Se film precursor annealed at 875 K for 5 min and Si were given as compared. All hyperdoped Si samples were fabricated with a 4.0 kJ/m^2^ laser fluence.

Figure 3 shows the Raman spectra obtained from the hyperdoped samples and the planar Si reference. The characteristic crystalline Raman peaks (~303 and ~519 cm^−1^) and the broad amorphous Si (a-Si) peaks (~150, ~300, and ~470 cm) are visible for all the hyperdoped Si samples compared to the crystalline Si reference. From the similar spectra of all the hyperdoped Si samples, it can be inferred that the crystallinity of Si:N/Se samples is insensitive to the thickness of the precursor Se films. The similar Raman spectrum after the RAT process further indicates excellent crystallinity and thermal stability. Although the amorphous structures indicated by Raman spectra leave room for further material optimization, the good crystallinity and high absorption indicate promising photoelectric performance.

**Figure 4:**
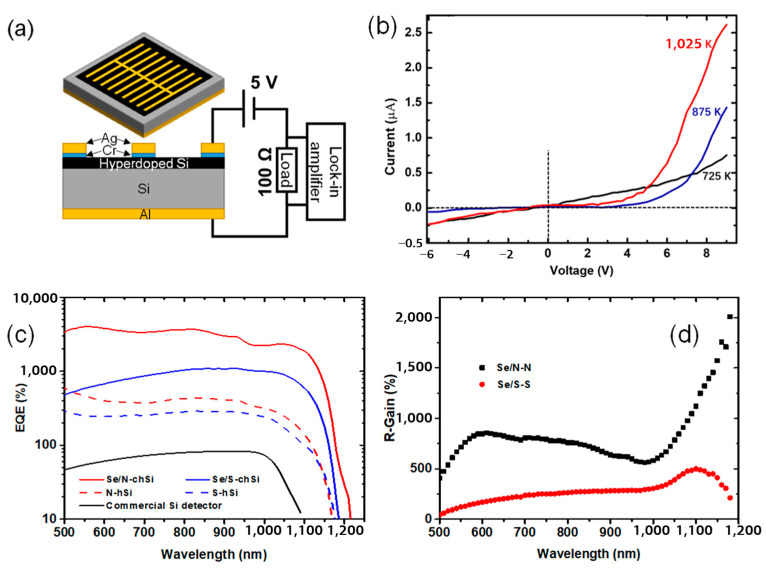
(**a**) The schematic structure of hyperdoped Si photodetectors and the photoelectric responsivity measurement system. (**b**) The I-V characteristics of the photodetectors based on Si:N/Se with RAT at 725 K, 875 K and 1025 K for 5 min. (**c**) Spectral photoelectric responsivity of hyperdoped Si photodetectors and a commercial silicon photodetector used for calibration for comparison. (**d**) Responsivity gain (R–G) factors of the Si:N/Se vs. Si:N and Si:S/Se vs. Si:S.

Figure 4a illustrates the structure of photodetectors fabricated from our hyperdoped Si samples and the measurement setup. The front comb-like Cr/Ag (20/400 nm) contact and rear Al (400 nm) contact on the hyperdoped Si and commercial Si were deposited using E-beam evaporation. Here, all hyperdoped Si samples were prepared in the fluence of 4.0 kJ/m^2^ (coated with 100 nm Se films) to ensure the proper surface morphology (with a typical spike height of 3–4 μm) for the electrode contact. However, the optical absorptance displayed in Figure 2b,c is slightly lower than that of 5.6 kJ/m^2^. Following metal deposition, a rapid thermal annealing process at 625 K for 1 min in a protective gas atmosphere (N_2_/H_2_, 95%/5%) flow was applied to improve the contacts.

The I-V characteristics of these photodetectors were measured using a semiconductor parameter analyzer (4200-SCS/F); as seen in Figure 4b, higher RAT temperature (up to 1025 K) induces better rectification properties as inferred from higher current at positive bias. Previous studies have identified 975 K (700 °C) as the optimal annealing temperature for improving the optical absorption, rectifying behavior, and photoresponsivity for Si:Se samples. In this work, more devices were fabricated based on hyperdoped Si annealed at 1025 K to study the photoelectric characteristics. In addition to devices based on hSi fabricated with an NF_3_ atmosphere, samples with SF_6_ atmosphere were also analyzed, i.e., Si:S and Si:S/Se, for comparison. A photoelectric measurement system composed of a bromine tungsten lamp (LSH-T150, 150 W), a grating monochromator (Omni-λ 300, cover 400–2500 nm with 0.1 nm spectral resolution), and a lock-in amplifier (Stanford SR830) was used to measure the photoelectric response characteristics at room temperature in a dark environment. During the measurement, the lock-in amplifier parallel with a load resistance (100 Ω) and a 5 V bias was added on all these photodetectors including the commercial Si planar, as exhibited in Figure 4a.

The measured responsivity spectra are shown in Figure 4c. All hyperdoped Si photodetectors exhibit much (1–2 orders of magnitude) higher responsivity than the commercial Si photodetectors in the 500–1100 nm wavelength range. The Si:N/Se photodetector shows remarkable photoresponsivity of 24.8 and 19.8 A/W at 840 and 1060 nm, respectively. The responsivity of the photodetector R could be described as $R_{\lambda }=\frac{I_{\lambda }-I_{d}}{P_{\lambda }S}$, where *I_λ_* is the photocurrent, *I_d_* is the dark current, *P_λ_* the pumping intensity, and *S* is the effective illuminative area on the device [27]. These correspond to the external quantum efficiency (EQE) of 3661% and 2316%, respectively. Without the Se precursor, the Si:N photodetector shows one-order-of-magnitude lower responsivity (only 2.9 A/W and 2.1 A/W, respectively). Similarly, for samples prepared in SF_6_ atmosphere, the Si:S/Se photodetector shows notably higher responsivity than its Si:S counterpart (e.g., 7.6 vs. 1.4 A/W at 1060 nm). More carriers for the Se co-doped with S and N synergically enhanced the photoconductivity [28,29]. Se/N and Se/S formed different complexes in these hSi samples, and the complexes induced by Se/N had reduced structural defects and released more electrons [12,20].

To see the synergic effect of co-hyperdoped elements on the photoelectric performance, we define a responsivity gain (G) factor as $G=\frac{R_{1}}{R_{2}}-100\%$. For hyperdoped samples fabricated in NF_3_, *R_1_* and *R_2_* are the photodetector’s responsivity in Si:N/Se and Si:N, respectively. When G > 0, it means the responsivity in Si:N/Se is larger than that in Si:N. For samples fabricated in SF_6_, *R_1_* and *R_2_* are that of Si:S/Se and Si:S, respectively. As compared in Figure 4d, the G factor in Si:N/Se is at least several times higher than that of Si:S/Se. A sharp increase in the G factor at wavelengths larger than 1000 nm is obtained, corresponding to the clear red shift in the response tail in the Si:N/Se photodetector. For the sample of Si:S/Se, the G factor is much lower. This indicates that Se has a better synergic effect with N than that with S.

In addition, in the wavelengths beyond the silicon bandgap, the hyperdoped materials showed strong optical adsorption and relatively low photoresponsivity. Many researchers have been dedicated to achieving an ideal result: high optical absorption should lead to effective photoresponsivity, but there is still no high infrared photoresponsivity. The DFT calculation and analysis indicated that the defect (Se/N/S) structures and disordered impurities jointly decided the concentration and intermediate band (IB) level, which further influenced the absorption and photocarriers [30,31,32]. Among many doping defects, many Se/N/S defects would not lead to an increased photocurrent. Therefore, they cannot contribute to the photocarriers, especially for lower energy defects. Further research on the defect structures for S/Se and N/Se co-doped in silicon will be presented in our later report.

## 4. Conclusions

In this study, the Si:N/Se samples demonstrate excellent crystallinity, which contributes to low carrier recombination within the hSi materials, thermally stable absorption, and enhanced responsivity across an extended wavelength range. Following annealing at 1025 K for 5 min, the photoresponse of the Si:N/Se diodes increased by one to two orders of magnitude compared to the Si:N, Si:S, and Si:S/Se diodes in the wavelength range of 500 to 1100 nm, achieving corresponding external quantum efficiencies (EQEs) of 3661% and 2316%, respectively. However, further precise characterization at longer wavelengths is necessary, as the photoelectrical signal remains significantly lower than that observed in the above-bandgap range. Improved photoelectric performance is anticipated with additional optimization and enhancements in material properties, such as the incorporation of tellurium (Te) as a precursor, as well as improvements in surface passivation and contact quality. The approach of co-hyperdoping with various elements provides a flexible strategy for enhancing responsivity across a broad wavelength range, from the visible to the infrared spectrum. Moreover, exploring additional combinations of co-doped elements may soon further enhance the performance of silicon-based detectors.

## Data Availability

The raw data supporting the conclusions of this article will be made available by the authors on request.
